# Cultural adaptation of WHO's iSupport program for dementia carers in Vietnam: the e‐DiVA Vietnamese component

**DOI:** 10.1002/alz70858_097770

**Published:** 2025-12-24

**Authors:** Thanh Binh Nguyen, Trung Anh Nguyen, Thanh Binh Nguyen, Anh Nguyen Ngoc, Binh Nguyen Thi Thanh, Anh Nguyen T Phuong, Phong Nguyen Quy, Thang Pham, Thu Ha Dang, Tuan Anh Nguyen

**Affiliations:** ^1^ National Geriatric Hospital, Hanoi, Viet Nam; ^2^ Hanoi Medical University, Hanoi, Viet Nam; ^3^ Swinburne University of Technology, Melbourne, VIC, Australia; ^4^ La Trobe University, Melbourne, VIC, Australia; ^5^ National Ageing Research Institute, Melbourne, VIC, Australia; ^6^ University of South Australia, Adelaide, SA, Australia

## Abstract

**Background:**

Supporting carers is a key priority in the Global Action Plan on Dementia. The World Health Organization (WHO) developed iSupport, an online education and support program for family carers of people with dementia that can be adapted for use in different contexts. This study aimed to adapt iSupport for Vietnamese dementia carers.

**Method:**

The adaptation process followed the WHO iSupport Adaptation and Implementation Guidelines in two stages. **Stage 1** involved forward translation, expert panel review, backward translation, and harmonization, followed by researcher‐led adjustments to align the content with Vietnamese cultural and healthcare contexts. **Stage 2** consisted of Focus Group Discussions (FGDs) with family carers and healthcare professionals (HCPs) to gather feedback on the program's content and its delivery via videos and an online virtual assistant platform. Data were analyzed thematically.

**Result:**

In **Stage 1**, professional translations were refined to ensure alignment with Vietnamese medical terminology, cultural expressions, and caregiving practices. Adjustments included replacing unfamiliar activities with culturally relevant ones and adapting medical advice to Vietnamese standards, such as consulting dentists instead of general practitioners for oral health issues. Vietnamese proverbs, idioms, and localized names were incorporated to enhance cultural resonance.

**Stage 2** generated valuable feedback through FGDs. Participants emphasized the need for simplified, jargon‐free language and recommended practical, context‐specific caregiving tips, such as breaking water intake into smaller portions to prevent dehydration. They strongly favoured digital delivery methods for the program, citing accessibility and convenience, with preferences for concise multimedia videos featuring real actors.

Participants appreciated iSupport's reliable and curated information, contrasting it with the overwhelming and often unreliable online resources. However, key barriers included limited time availability among carers and the need for stage‐specific guidance tailored to the progression of dementia. Participants suggested interactive features, such as notifications for updates, to enhance usability.

**Conclusion:**

The study underscores the potential of the iSupport program to address gaps in dementia care knowledge and support among Vietnamese carers. By incorporating culturally tailored content and leveraging accessible digital platforms, iSupport can significantly improve caregiving practices and outcomes in Vietnam.

